# Risk factors, presentation and outcome of meningomyelocele repair

**DOI:** 10.12669/pjms.36.3.1237

**Published:** 2020

**Authors:** Lal Rehman, Munwar Shiekh, Ali Afzal, Raza Rizvi

**Affiliations:** 1 Dr. Lal Rehman, FCPS. Department of Neurosurgery, Jinnah Postgraduate Medical Center, Karachi, Pakistan; 2 Dr. Munwar Sheikh, FCPS. Department of Neurosurgery, Jinnah Postgraduate Medical Center, Karachi, Pakistan; 3 Dr. Ali Afzal, FCPS. Department of Neurosurgery, Jinnah Postgraduate Medical Center, Karachi, Pakistan; 4 Dr. Raza Rizvi, MS. Department of Neurosurgery, Jinnah Postgraduate Medical Center, Karachi, Pakistan

**Keywords:** Meningomyelocele, Neural Tissue, Maternal folate intake, MMC repair

## Abstract

**Objective:**

To determine the risk factors, presentation and outcome of meningomyelocele repair

**Methods:**

We reviewed 150 cases operated for meningomyelocele (MMC) at Jinnah Postgraduate Medical Centre Karachi between May 2015 and May 2018. Data of infants operated for MMC repair was extracted including socioeconomic status, maternal folate intake during pregnancy, head circumference, location and width of the defect, accompanying bladder and limb anomalies and treatments administered. Patients were followed up for a mean period of six months.

**Results:**

A total of 150 children were evaluated, out of which there were 83(55.3%) males and 67(44.7%) females. All belonged to low socio economic group and prenatal maternal folate intake as risk factor was positive in 103(68.7%) cases. Mean head circumference was 37.4 cm (range, 30.7 to 50 cm). Based on their location, 83(55%) of the defects were lumbosacral, 38(25.4%) were lumbar, 16(10.7%) were thoraco lumbar, 10(6.7%) were thoracic and three (2%) were cervical. Mean size of the meningomyelocele sac was 4.3 cm×5.6 cm (range, 1cm×2 cm to 11cm×8.4cm) and 21(14%) of the babies had a skin defect requiring flap. According to accompanying anomalies, 98(65.3%) of the babies had hydrocephalus, 13(9%) had club foot, four (2.7%) had diastematomyelia and three (2%) had tethered cord. Eighty seven (58%) patients had neurological deficit pre operatively and eight (5.4%) patients with normal power deteriorated after surgery out of which five (3.3%) developed paraplegia and three (2%) developed paraparesis. CSF leak was the major complication encountered in 16(11%) followed by meningitis in seven (5%), while the overall mortality was four (2.6%).

**Conclusion:**

The practice of periconceptional folic acid supplementation is essential to reduce the prevalence of neural tube defects (NTDs) in the developing world. Improved maternal nutrition with access to quality antenatal care is vital to decrease the prevalence and health burden.

## INTRODUCTION

A potentially preventable cause of perinatal morbidity and mortality, NTDs are one of the most common congenital malformations. The incidence is one in every 800–1000 live births^1^ which varies according to the geographic conditions, race, sex of the baby and certain maternal conditions. Studies in Pakistan estimate incidence between 38.6 and 124.1 per 10,000 births^2^,^3^ The mortality rate in the first 6 months of life is 65%–70% in untreated patients.^4^ This congenital anomaly of the central nervous system (CNS) affects the neural tube in the early phases of neurulation in the 3rd or 4th week of development. Based on whether the rostral or caudal neuropore fails to close, NTDs are classified into cranial dysraphism with resultant anencephaly or spinal dysraphism with or without MMC.

In MMC, a part of the spinal cord, together with the surrounding meningeal structures, herniates outward through the defective bony arches and skin as a sac. The protrusion of the meninges and spinal cord through open vertebral arches is often associated with paralysis and varying degrees of mental retardation, bowel and bladder dysfunction as well as orthopedic disabilities.^5^ The greater the amount of neural tissue inside the sac, the worse is the neurologic deficit and prognosis. It is important to close MMC defects in the early postnatal period to decrease mortality rates by providing protection for neural elements and preventing CSF leakage and related central nervous system infections.^6^ MMC repair is a relatively uncommon procedure in the Western world secondary to better nutrition and early detection but it still poses a significant health burden in the developing world with devastating outcomes for the affected child and family.

Folic acid deficiency is one of the best documented risk factors for the development of NTD’s.^7^ Hydrocephalus accompanies MMC in nearly 80% of all cases followed by other systemic anomalies in varying order.^4^ MMC’s are classified by location with the most common site being lumbar followed by the lumbosacral region. Treatment involves early closure of the neural tissue, repair of the skin defect, and placement of ventriculoperitoneal (VP) shunt in cases with accompanying hydrocephalus. Most MMC defects can be closed primarily, but this may not be possible in up to 25% of cases.^6^ In this study we present our institutional experience of risk factors, presentation and outcomes of MMC repair.

## METHODS

We reviewed 150 cases operated for MMC between May 2015 and May 2018. Data of infants operated for MMC repair was extracted including socioeconomic status, maternal folate intake during pregnancy, head circumference, location and width of the defect, accompanying bladder and limb anomalies and treatments administered. Consecutive patients with MMC aged ≤1year, with and without hydrocephalus were included while children having other systemic anomalies, leaking MMC, severe kyphotic deformity, very small MMC with HCP treated with shunt alone and those who had been operated elsewhere were excluded. A multidisciplinary approach was undertaken that included pediatrician, urologist, orthopedic and plastic surgeons. After repair, infants were referred to respective specialty for further treatment and follow up as well. For very large defects, plastic surgery was taken on board for construction of flaps for proper closure. Data was analyzed using SPSS version 23 and the data were expressed as mean ± SD (standard deviation) and percentage (%), as appropriate.

## RESULTS

**Fig.1:**
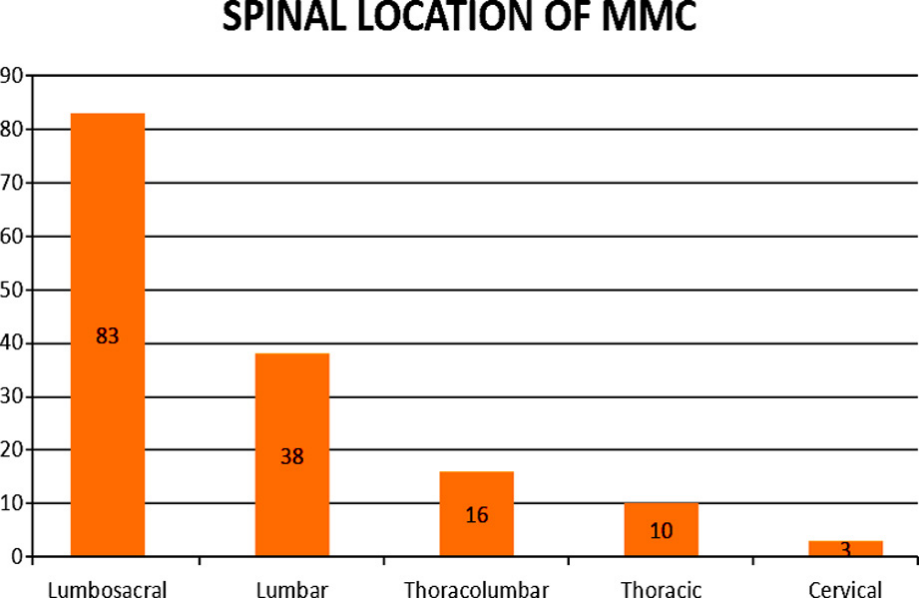
Location.

**Fig.2:**
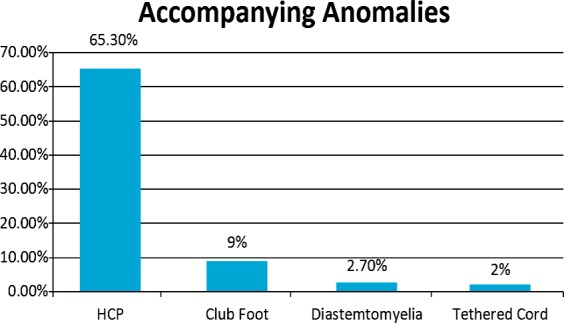
Associated Anomalies.

A total of 150 children were evaluated out of which there were 83(55.3%) males and 67(44.7%) females. All belonged to low socio economic group and prenatal maternal folate intake as risk factor was positive in 103(68.7%) cases. Mean head circumference was 37.4 cm (range, 30.7-50 cm). Based on their location, 83 (55%) of the defects were lumbosacral, 38(25.4%) were lumbar, 16(10.7%) were thoraco lumbar, 10(6.7%) were thoracic and three (2%) were cervical as shown in Fig.1. Mean size of the meningomyelocele sac was 4.3 cm×5.6 cm (range, 1cm×2 cm to 11cm×8.4cm) and 21(14%) of the babies had a skin defect requiring flap. According to accompanying anomalies, 98(65.3%) of the infants had hydrocephalus, 13(9%) had club foot, four (2.7%) had diastematomyelia and three (2%) had tethered cord as shown in Fig.2. Eighty seven (58%) patients had neurological deficit pre operatively and eight (5.4%) patients with normal power deteriorated after surgery out of which five (3.3%) developed paraplegia and three (2%) developed paraparesis. CSF leak was the major complication encountered in 16(11%) followed by meningitis in seven (5%) while the overall mortality was four (2.6%).

## DISCUSSION

The second most frequently encountered congenital malformations after cardiac anomalies are NTDs and folic acid deficiency is the most widely recognized risk factor which is more common in the low socioeconomic group.^8^ Similar findings are reported by studies in the developing world which found NTDs in low socioeconomic groups.^9^ Preconceptional supplementation of folic acid data from a supplementation program in China suggest that folic acid interventions can reduce NTD prevalence to as low as 5–6 per 10,000 pregnancies 10 which highlights the importance of antenatal folic acid supplementation for prevention. Most of the mothers of the subjects included in this study had not used any form of folic acid supplementation either before or during their pregnancies. Prenatal maternal folate intake as risk factor was positive in 103(68.7%) cases which is lower in comparison to a study from Ethopia by that report only 14.4% of the participants had preconception folic acid or multivitamin supplementation.^11^

The mean age of presentation in our patients was 2 months ±1SD similar to a recent study by Alamgir^12^, where average age of presentation was 58.58 ± 26.01 days. There were 83(55.3%) males and 67(44.7%) females in our study which relates to 56.4% male and 43.6% female incidence in the Alamgir study from a similar geographical area. The most common site of MMC occurs in the lumbar area, with a reported frequency of 60-70%.^13^ The mean head circumference was 37.5 similar to a Turkish study by Oncel 14 where mean head circumference was 35.8±3.8 cm. In our study, 83 (55%) of the defects were lumbosacral and 38(25.4%) were lumbar that is similar to results of Oncel^14^ who reported 46.6% of the defects were lumbosacral and 40% were lumbar. Mean size of the MMC sac was 4.3 cm×5.6 cm (range, 1cm×2 cm to 11cm×8.4cm) which is similar to Alamgir^12^ where 79 patients had a defect size < 5 cm. 21(14%) of the infants had a skin defect requiring flap which is consistent with findings in literature where in most MMC defects, primary closure was achievable but this may not be possible in up to 25% of cases.^6^

Hydrocephalus and the Chiari II malformation were the most frequently observed anomalies in association with MMC.^15^ In our study, hydrocephalus was present in 98(65.3%) of the infants, 13(9%) had club foot, four (2.7%) had diastematomyelia and three (2%) had tethered cord. Other studies have also found comparable incidence of hydrocephalus requiring CSF diversion during the same hospital stay like Kshettry^16^ where 56.6% of patients required shunt placement. Therefore, it is necessary that all patients with MMC, in addition to a detailed and careful physical examination, should be screened for the presence of other anomalies like cranial and cardiac imaging studies as well as urinary system ultrasonography.^17^,^18^ Early surgery has been shown to be associated with lower morbidity and mortality^19^ and one study demonstrated that surgery immediately after birth has lesser risks and superior outcomes compared to fetoscopic surgery that has significant risks for both the mother and the fetus.^20^

The major complication of CSF leak was encountered in 16(11%) followed by meningitis in seven (5%) which is similar to infection rates in a series by Demir at 11%^21^ but lower in comparison than a similar local study reporting wound site infection in 13.5% patients and a high number of CSF leak in 23.7% patients where most of these patients required CSF diversion eventually.^22^ Eighty seven (58%) patients had neurological deficit pre operatively and eight(5.4%) patients with normal power deteriorated after surgery out of which five(3.3%) developed paraplegia and three(2%) developed paraparesis. The overall mortality in our series was four (2.6%) secondary to meningitis and respiratory compromise similar to Kshettry^17^ where the in-hospital mortality rate was 1.4%.

There were no other complications till last follow up. However, spinal cord rethetering can occur after the primary surgical repair of MMC and inadvertent introduction of skin elements at first surgery can lead to the growth of intraspinal epidermoid or dermoid cysts.^22^ So, a close and continuous follow up is mandated in each case. Increased public awareness, prevention, quality antenatal surveillance and early surgical intervention are also factors which contribute to better prognosis. It is recommended to consider implementing national preventive strategies to reduce the prevalence of NTDs in our country and the developing world along with developing a national database for establishing guidelines and timely referrals for improved outcomes.

## CONCLUSION

The practice of routine periconceptional folic acid supplementation is negligible and improved maternal nutrition with access to quality antenatal care is vital to decrease the incidence and health burden of this devastating disease. Early surgery with multidisciplinary approach offers the best chance for improved outcomes and survival.

### Authors Contribution:

**MS:** Conceived and designed the study.

**AA:** Did data collection and manuscript writing.

**RR:** Did statistical analysis & editing of manuscript.

**LR:** Did review, final approval of manuscript and is responsible for integrity of research.
